# Coupled ferroelectric polarization and magnetization in spinel FeCr_2_S_4_

**DOI:** 10.1038/srep06530

**Published:** 2014-10-06

**Authors:** L. Lin, H. X. Zhu, X. M. Jiang, K. F. Wang, S. Dong, Z. B. Yan, Z. R. Yang, J. G. Wan, J.-M. Liu

**Affiliations:** 1 Laboratory of Solid State Microstructures, Nanjing University, Nanjing 210093, China; 2 Institute for Quantum Materials, Hubei Polytechnic University, Huangshi 435100, China; 3 Department of Physics, Southeast University, Nanjing 211189, China; 4 Institute of Solid State Physics, Chinese Academy of Sciences, Hefei 230031, China

## Abstract

One of the core issues for multiferroicity is the strongly coupled ferroelectric polarization and magnetization, while so far most multiferroics have antiferromagnetic order with nearly zero magnetization. Magnetic spinel compounds with ferrimagnetic order may be alternative candidates offering large magnetization when ferroelectricity can be activated simultaneously. In this work, we investigate the ferroelectricity and magnetism of spinel FeCr_2_S_4_ in which the Fe^2+^ sublattice and Cr^3+^ sublattice are coupled in antiparallel alignment. Well defined ferroelectric transitions below the Fe^2+^ orbital ordering termperature *T_oo_* = 8.5 K are demonstrated. The ferroelectric polarization has two components. One component arises mainly from the noncollinear conical spin order associated with the spin-orbit coupling, which is thus magnetic field sensitive. The other is probably attributed to the Jahn-Teller distortion induced lattice symmetry breaking, occuring below the orbital ordering of Fe^2+^. Furthermore, the coupled ferroelectric polarization and magnetization in response to magnetic field are observed. The present work suggests that spinel FeCr_2_S_4_ is a multiferroic offering both ferroelectricity and ferrimagnetism with large net magnetization.

For multiferroic applications, one of the most desirable and appealing functionalities is the coexisting ferroelectricity and ferromagnetism as well as their strong coupling^1^,^2^. This is the main motivation driving intense researches in the past decade. The unusual coupling between the two types of ferroic orders has been paid attention since the pioneer works on BiFeO_3_ and TbMnO_3_^3^,^4^. Nevertheless, so far discovered multiferroics manifest diverse crystallographic structures and ferroelectric origins. The most concerned multiferroics includes (1) perovskite RMnO_3_ (R = Dy, Tb, Gd, Eu_1-*x*_Y*_x_*)^5^,^6^, hexaferrites Ba_2_Mg_2_Fe_12_O_22_^7^, and LiCu_2_O_2_^8^, where noncollinear spin orders and spin-orbit coupling (SOC) play the central roles; (2) orthorhombic RMnO_3_ (R = Ho, Y)^9^ and rhombohedral Ca_3_CoMnO_6_ with collinear spin orders and spin-lattice coupling^10^; and (3) orthorhombic RMn_2_O_5_ (R = Tb, Ho, Dy) with coexisting noncollinear and collinear orders, etc^11^.

Unfortunately, a common characteristic of these multiferroics is the antiferromagnetically induced ferroelectricity. In other words, no matter how the SOC or the spin-lattice coupling as the microscopic mechanism for the ferroelectricity generation works, the antiferromagnetic (AFM) order is the pre-requisite in addition to the sufficient band gap. Therefore, coexistence of ferroelectricity and ferromagnetism remains to be a substantial issue so far. A well known exception is CoCr_2_O_4_ in which not only the AFM-induced ferroelectric (FE) polarization (*P*) but also large net magnetization (*M*) was observed besides the strong magnetic control of polarization reversal^12^,^13^. The reason is that the Cr^3+^ spins order in a conical geometry so that a net but yet weak magnetization is available. To our best knowledge, CoCr_2_O_4_ has probably been the only multiferroic offering well defined single-loop *P-H* and *M-H* hysteresis where *H* is magnetic field, due to the conical spin structure developed below temperature *T* ~ 26 K^12^.

CoCr_2_O_4_ belongs to the well known spinel (magnetite) family with AB_2_X_4_ formula, where A = (Fe, Co, Mn, Cu, Hg), B = (Cr, V, Sc), and X = (O, S, Se)^14^,^15^. The unusual multiferroic behaviors of CoCr_2_O_4_ have motivated further investigations on the magnetism and electronic properties of this spinel family in the past years. This family, however, exhibits diverse physical phenomena, including magnetoresistance associated with the Verwey transitions^16^, spin-orbital liquid (FeSc_2_O_4_) and spin liquid (MnSc_2_O_4_)^17^, orbital glass (FeCr_2_S_4_)^18^, and colossal magnetocapacitance (CdCr_2_S_4_, HgCr_2_S_4_)^19^,^20^. The interplay of spin, orbit, and lattice degrees of freedom is believed to play an important role in driving these emergent phenomena. For the multiferroicity, CdCr_2_S_4_ is one of the few compounds besides CoCr_2_O_4_ that ferromagnetic (FM) order weakly couples with relaxor ferroelectricity^19^. The relaxor dipole was claimed to originate from the off-center position of Cr^3+^ ion^21^. Furthermore, quite a number of members in this family exhibit the ferrimagnetic (FIM) structure with considerable SOC. The FIM lattice allows a nonzero *M* even though the nature of interactions is AFM. If such an AFM order favors ferroelectricity, the coexistence of nonzero *P* and *M* in such FIM lattice becomes accessible, so long as the two magnetic sublattices have non-equivalent moments. This is the motivation for searching candidate multiferroics with emergent coupling of FE and FM orders.

In this work, we address the magnetic and electrical properties of FeCr_2_S_4_ which is also a FIM insulator at low *T*^22^. A choice of FeCr_2_S_4_ is based on earlier works on the lattice/electronic structures and magnetodielectric behaviors^22^,^23^,^24^. First, as shown in Fig. 1(a) and (b), Cr^3+^ ions (3*d*^3^, *S* = 3/2) occupy the octahedral sites and the Cr^3+^-Cr^3+^ pairs are dominated with the FM exchange via the 90° Cr^3+^-S^2−^-Cr^3+^ bond angle, while the Fe^2+^ ions (3*d*^6^, *S* = 2) are located at the tetrahedrally coordinated sites. The exchange interaction within the Fe sublattice is weak and overruled by the much stronger AFM Fe^2+^-Cr^3+^ exchange, leading to the FIM ordering with interactive Cr^3+^ and Fe^2+^ sublattices at temperature *T* = *T_N_*~ 170 K. A cubic to triclinic phase transition at *T* ~ 60 K was once reported but far from confirmed in other experiments^25^. Below *T* ~ 60 K, the orbital moment of Cr^3+^ ions is quenched due to the half-filled *t_2g_* ground state until an orbital ordering at *T_oo_* = 8 ~ 10 K which, however, arises from the orbital ground state of Fe^2+^ ions with unfilled *e_g_* state rather than Cr^3+^ ions^18^. For the Fe^2+^ ions here, the spin degeneracy is lifted and splits into 5 levels by the internal exchange field^23^. This degeneracy can be further coupled to the lattice vibrations, leading to a cooperative Jahn-Teller (JT) distortion occurring at *T_oo_*, a critical event^26^. It is noted that in the orbital physics, usually the strong electron-phonon coupling can lift the orbital degeneracy and results in a long-range orbital ordering (OO) which lowers the crystal symmetry via the JT effect^27^. Quite a few theoretical models were proposed to correlate the ferroelectricity with such JT distortion and OO structure, mainly in ABO_3_-type perovskite oxides^28^.

Nevertheless, available data on the lattice and spin structures of FeCr_2_S_4_ are authors-dependent. Earlier neutron powder scattering at 4.2 K claimed the simple cubic spinel structure and the collinear FIM spin order^29^,^30^, however, recent neutron scattering indicated that the maximum displacement of the ligand S^2−^ ions is estimated to be ~0.04Å, and the resultant splitting of the Bragg reflection could not be resolved^24^. Muon spin rotation/relaxation (μSR) spectroscopy indicated that the collinear FIM structure evolves into an incommensurately modulated noncollinear spin structure below ~50 K, which then remains stable down to the Fe^2+^ orbital ordering *T_oo_*^31^. It seems that two antiferromagnetically coupled cone-like spiral spin structure sets in *T* ~ 50 K, and an OO sequence due to the JT effect enters at *T_oo_*. The favored story is that the former contributes to a net magnetization and the latter to a possible electric polarization. Consequently, the Cr^3+^ and Fe^2+^ sublattices with noncollinear moments are coupled in a roughly anti-parallel alignment, leading to weak structural distortion as revealed by a series of measurements including the X-ray synchrotron powder diffraction, sound-velocity, and thermal-expansion^23^.

Certainly, the FIM state is highly favored here for multiferroicity because it allows a net magnetization, a major step towards the ferromagnetism. On the other hand, the strong electron-phonon coupling and the Fe^2+^ orbital ordering suggest a potential for magnetically induced ferroelectricity^32^. Recent dielectric permittivity data indicate remarkable magnetodielectric response although no details of the underlying physics have been clarified^24^. In this work, we pay our attention to the low-*T* pyroelectric current measurements of FeCr_2_S_4_ in addition to other structural and magnetic characterizations. Our results provide clear evidence that FeCr_2_S_4_ does show a FE transition at *T_oo_* and a coexistence of nonzero electrical polarization and nonzero magnetization. And more importantly, FeCr_2_S_4_ exhibits strong magnetoelectric coupling, and the ferroelectric mechanism is subsequently proposed based on the relevant data.

## Results

### Specific heat & magnetic properties

**Figure 1:**
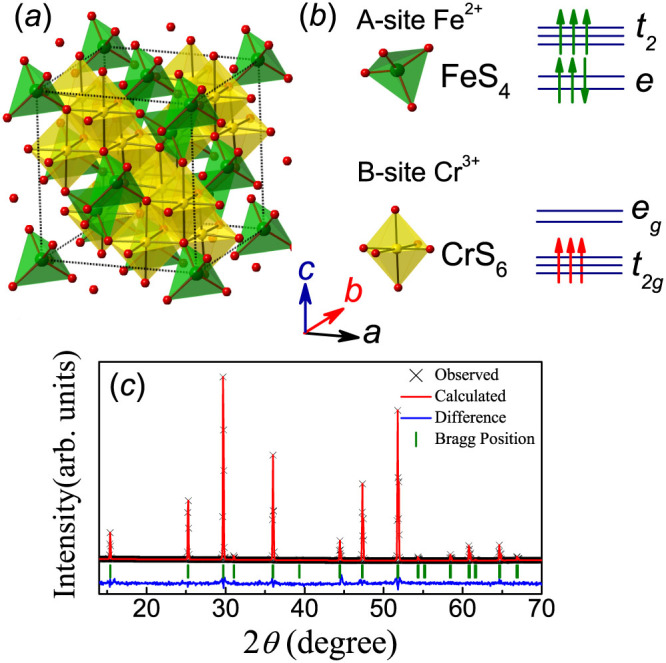
(a) A schematic crystal structure of FeCr_2_S_4_. The Fe^2+^ and Cr^3+^ ions are located at the center of tetrahedral and octahedral S^2-^ cages, respectively. (b) Occupation of the crystal-field levels for Fe^2+^ and Cr^3+^ in FeCr_2_S_4_. (c) The XRD spectra and Rietveld refinement of powder FeCr_2_S_4_ at room temperature.

Before presenting the specific heat and magnetic data, we first identify the crystallinity at room temperature and the XRD *θ*-2*θ* spectrum is plotted in Fig. 1(c). All the reflections are well indexed by single phase cubic FeCr_2_S_4_ and no impurity phase is detected. We perform the Rietveld refinement of the XRD data using the GSAS program^33^, and the refined result is in good agreement with the cubic structure of lattice constant *a* = 9.9920Å, with reliability parameters *R_wp_* = 5.78%, *R_p_* = 4.23%, and *χ^2^* = 2.25.

**Figure 2:**
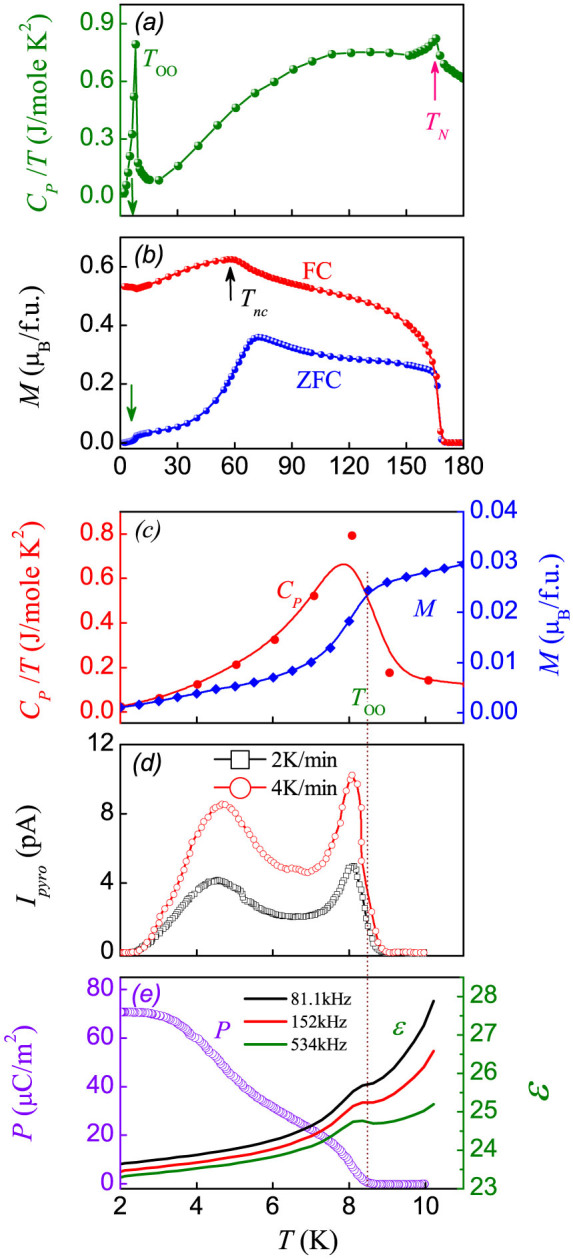
(a) Specific heat *C_p_*/*T* vs *T* plot, showing the ferrimagnetic and orbital ordering transitions at *T_N_* and *T_oo_*. (b) *T* dependence of *M* in the ZFC and FC modes. (c) The *C_p_* and *M* within *T* = 2–11 K. (d) Measured *I_pyro_* as a function of *T* under different warming rate (2 and 4 K/min). (e) *T* dependence of polarization (*P*) and dielectric constant (*ε*) at three different frequencies as labeled.

The measured specific heat *C_p_*(*T*) data are presented in Fig. 2(a). Two clear anomalies in the *C_p_*(*T*) curve, respectively at *T_N_* = 165 K and *T_oo_* ~ 8.0 K are observed, which represent the FIM transition and Fe^2+^-OO transition, respectively. A weak bump at *T* ~ 60 K can be identified, which is attributed to the evolution of magnetic domain structure due to a structural transformation. These features finds the one-to-one correspondences in the *M*(*T*) curves under the ZFC and FC modes, as shown in Fig. 2(b). The sharp shoulder-like feature at *T_N_* is from the FIM transition, below which the *M* values in the ZFC and FC modes begin to separate until the lowest-*T*. Since the Fe^2+^ and Cr^3+^ spins have big difference in moment, the *M* can be as big as 1.6 *μ_B_*/*f*.*u*^24^. It should be mentioned that the separation in *M* between the ZFC and FC modes is attributed to the domain wall motion with pining centers, as previously reported^34^. On the other hand, the maximal *M* value around *T_nc_* for the FC mode is 0.62 *μ_B_*/*f.u.*, not only much smaller than either the moment of the Cr^3+^ sublattice or that of the Fe^2+^ sublattice, but also smaller than the net moment of the FIM state (~1.9 *μ_B_*/*f.u.*), assuming the collinear and antiparallel FIM lattice. This implies that the spins in the two sublattices align most likely in the noncollinear configuration, as suggested by the μSR spectroscopy^31^.

The *C_p_*(*T*) and ZFC *M*(*T*) data below *T* = 11 K are re-plotted in Fig. 2(c). The one-to-one correspondence between the *C_p_*-peak and the *M*-anomaly around *T_oo_* in the ZFC mode is seen, indicating the correlation between the Fe^2+^-OO transition and spin re-ordering. This spin re-ordering sequence is most likely due to the enhanced stabilization of the non-collinear spin configuration induced by the Fe^2+^-OO transition, noting that the non-collinear spin ordering is one of the pre-requisites for SOC driven ferroelectricity as identified in a number of multiferroics.

### Ferroelectric transitions

Now we focus on the dielectric and ferroelectric (FE) behaviors. The dielectric permittivity was applied by the parallel circuit mode, in which the accuracy of measurement is largely not affected by leakage current if any. In addition, the parallel equivalent circuit model can fit the data well by using Zview program. As expected, the dielectric constant falls continuously with decreasing *T* until an anomalous peak around *T_oo_*, as shown in Fig. 2(e). This anomaly signs a ferroelectric transition. It can be seen that there is no apparent shift of the anomalous peak over a broad range of frequency although normal dielectric dispersion behavior, featured by gradual decrease with increasing *f*, is observed. Then the measured *I_pyro_*(*T*) curves at two different rates are plotted in Fig. 2(d). Two clear *I_pyro_*-peaks are shown in each case and no identifiable *T*-axis shift of these peaks is seen, indicating that the *I_pyro_* does come from the pyroelectric effect. The evaluated polarization *P*(*T*) data from the two *I_pyro_*(*T*) curves fall on the master curve plotted in Fig. 2(e). The ferroelectric transition begins at *T_oo_* and the *P* reaches the saturated value of ~70 μC/m^2^ below *T* = 4 K. The saturated *P* is smaller than those magnetic multiferroics such as CaMn_7_O_12_^35^, and Tb(Dy)Mn_2_O_5_^11^ etc, but larger than the observed *P* for other spinel compounds like CoCr_2_O_4_^12^ and hexaferrites^36^.

It should be mentioned that the two *I_pyro_*(*T*) peaks most likely imply two ferroelectric transitions. The *P*(*T*) dependence confirms this argument. If the transition around *T_oo_* is related to the non-collinear spin order, the other (second) transition at lower *T* may possibly be the consequence of the JT distortion which lowers the lattice symmetry. If this argument applies, one expects that the electric polarization (*P_2_*) arisen from the transition around *T_oo_* is highly sensitive to magnetic field, and the polarization (*P_1_*) from the lower-*T* transition would be robust against magnetic field. Along this line, it is useful to investigate the response of electric polarization to magnetic field.

### Magnetoelectric response

**Figure 3:**
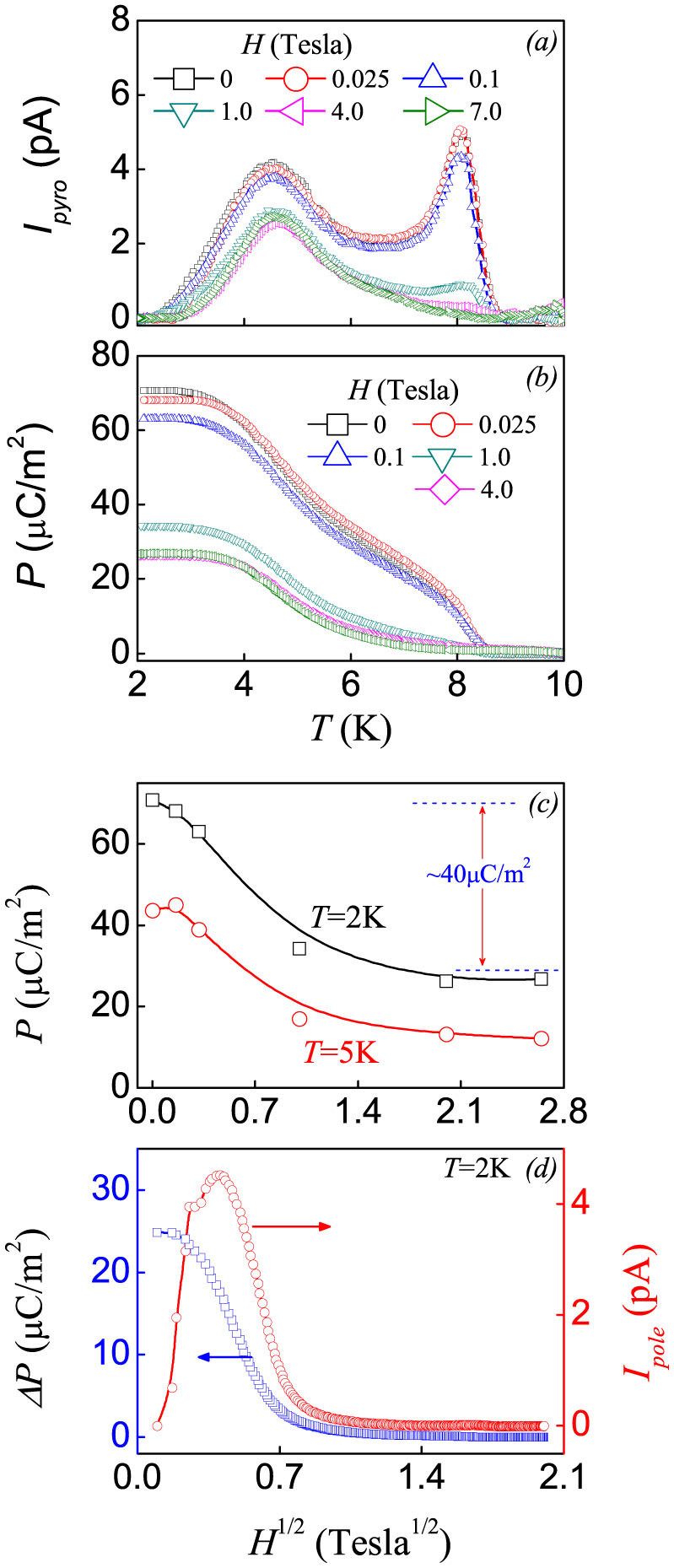
Measured *I_pyro_-T* data (a) and *P-T* data (b) under various magnetic fields. (c) Evaluated *P*-*H* relations under *T* = 2 K and 5 K. (d) Measured *I_pole_-H* and Δ*P-H* data at *T* = 2 K with scanning magnetic field rate 100 Oe/s.

We perform the iso-thermal and iso-field experiments on the magnetoelectric response. For the iso-field experiments, the *I_pyro_*(*T*) data under different magnetic fields *H* are plotted in Fig. 3(a) with the evaluated *P*(*T*) data presented in Fig. 3(b). As discussed, the prominent feature is the remarkable response of the *I_pyro_* peak around *T_oo_* to *H*, while the low *T* peak is much less changeable. The high *T* peak is totally suppressed at *H*>4.0 T, but the low *T* peak is only slightly suppressed under a field as high as 7.0 T. Besides, it is noted that the ferroelectric Curie point, as evaluated from the *P*(*T*) data, remains less affected either. We can plot the evaluated *P* data at *T* = 2 K and 5 K as a function of *H* in Fig. 3(c), noting here that the linear *H*-axis is re-scaled as *H*^1/2^ only for clear presentation of the data in the low-*H* range. The polarization is rapidly suppressed by increasing *H* in the low-*H* range but far from complete even under high *H*. At *T* = 2 K, the variation in *P* upon *H* up to 7.0 T is ~40 μC/m^2^, leaving the remnant polarization as big as ~30 μC/m^2^. Similar tendency is observed for the data at *T* = 5 K.

For the iso-thermal experiments, the *I_pole_*(*H*) data at *T* = 2 K are plotted in Fig. 3(d) together with the evaluated *ΔP*(*H*) data. The rapid suppression of *P* by *H* in the low-*H* range is featured by the *I_pole_*(*H*) peak and the rapid decay of *P* with increasing *H*. The *P* variation is ~25 μC/m^2^, slightly smaller than the iso-field data shown in Fig. 3(c). It should be mentioned that the *P*(*H*) data and the *ΔP*(*H*) data from the two different modes of measurement can't be identical, and finite differences between them are reasonable. Considering the measurement process, the captured current *I_pole_* should be purely considered as the induced magnetoelectric current (high-*T* peak) motivated by the magnetic field. However, for the pyroelectric current (*I_pyro_*-*T*) measurement mode, the *P*-*H* relation at 2 K shown in Fig. 3(c) should contain two components variation: the strong *H*-dependent high-*T* polarization, and relatively weak *H*-dependent low-*T* polarization.

### Two polarization components

**Figure 4:**
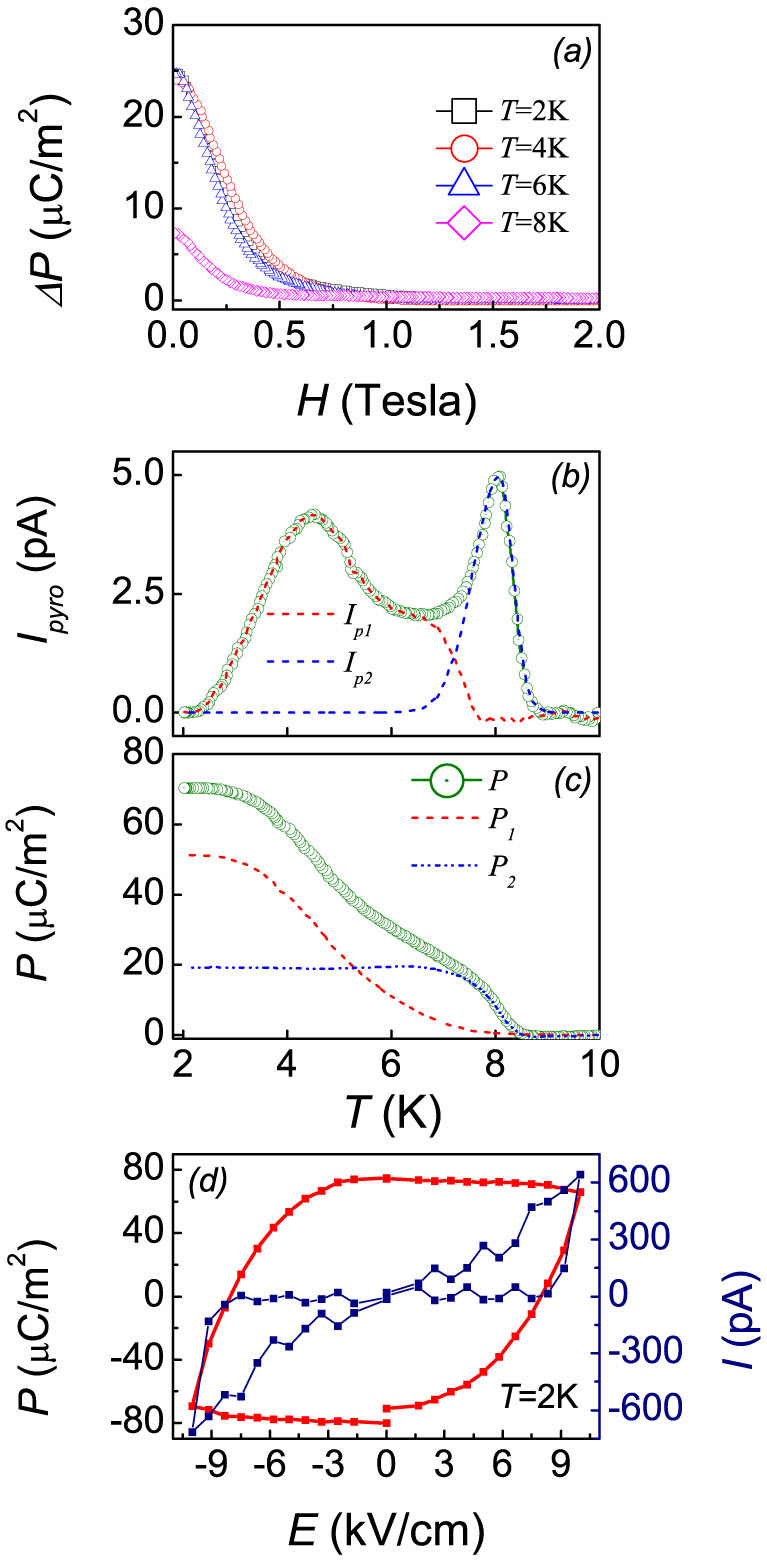
(a) *H* dependence of *ΔP* measured under *T* = 2 K, 4 K, 6 K, and 8 K. (b) Measured *I_pyro_* and the two separated single-peaked curves *I_p1_* and *I_p2_*. (c) *T* dependence of polarization *P* and the evaluated two components *P_1_* and *P_2_*, which correspond to the conical spin order and Jahn-Teller effect origin, respectively. (d) The *P*-*E* and *I*-*E* loop at *T* = 2 K obtained by PUND method.

The data presented above on ferroelectricity suggest the two polarization components which may have different origins. The magnetoelectric data allow a possible roadmap to separate the two components from each other. Indeed, the separation of the two components is far from accurate and it is impossible to give a convincing function to fit the *I_pyro_*(*T*) curve since we have no sufficient knowledge on the details of the FE polarization as a function of *T*. The high temperature current peak is supposed to come from the magnetoelectric domain via the inverse DM mechanism, which is seriously *H*-dependent. Here, we present the *ΔP*-*H* curves under different *T*, as shown in Fig. 4(a). The induced polarizations *ΔP* tuned by *H* varying from 0 to 2 Tesla at *T* = 2 K, 4 K, and 6 K are almost the same, implying that the stable conical spin order should be established below *T* = 6 K. Therefore, one reasonably assumes no more contribution from current *I_p2_* below 6 K, i.e. *I_p2_* = 0 at *T*<6 K. From this perspective, it should be practical to fit the high temperature contribution *I_p2_*. Here, we use Bigaussian function to fit this peak. The parameters for the Bigaussian function fitting are as follows: *y_0_* = 0, *x_c_* = 8.0784, *H* = 4.9826, *w_1_* = 0.50495, and *w_2_* = 0.25754. As shown in Fig. 4(b), the blue dashed curve (*I_p2_*) reflects the pyroelectric current contributed from the magnetically induced polarization which can be suppressed by *H*, whereas the red solid curve (*I_p1_*) reflects the pyroelectric effect arisen from the polarization which is only weakly *H*-dependent. The evaluated two components (*P_1_* and *P_2_*) as a function of *T* respectively are plotted in Fig. 4(c). It is seen that the FE transition associated with *P_2_* is relatively sharp and tends to be saturated. The transition associated with *P_1_*, however, is relatively diffused and *P_1_* can't be saturated until very low *T* (<3 K). The corresponding transition point is also slightly lower than that associated with *P_2_*.

To further explore the ferroelectricity in FeCr_2_S_4_, we also employ the Positive-Up-Negative-Down (PUND) method to measure the *P*-*E* loop. It is noted that PUND method is very effective to discriminate those undesirable factors (e.g., conductive and capacitance current) from the ferroelectric component, enabling the undesirable components to be subtracted without any assumption. Even though there are dramatic deviation between background and ferroelectric component, which may result in inaccurate polarization, the careful selection of amps range and good synchronization of the voltage step are much helpful to improve the accuracy of the data read-out. Fig. 4(d) shows the *I*-*E* and *P*-*E* loops measured under *T* = 2 K. The measured ferroelectric current is one order larger than the accuracy (~40 pA), implying the subtracted current data is still reliable. The PUND data confirms the ferroelectricity as revealed by the pyroelectric current method.

### Ferroelectric polarization and nonzero magnetization

**Figure 5:**
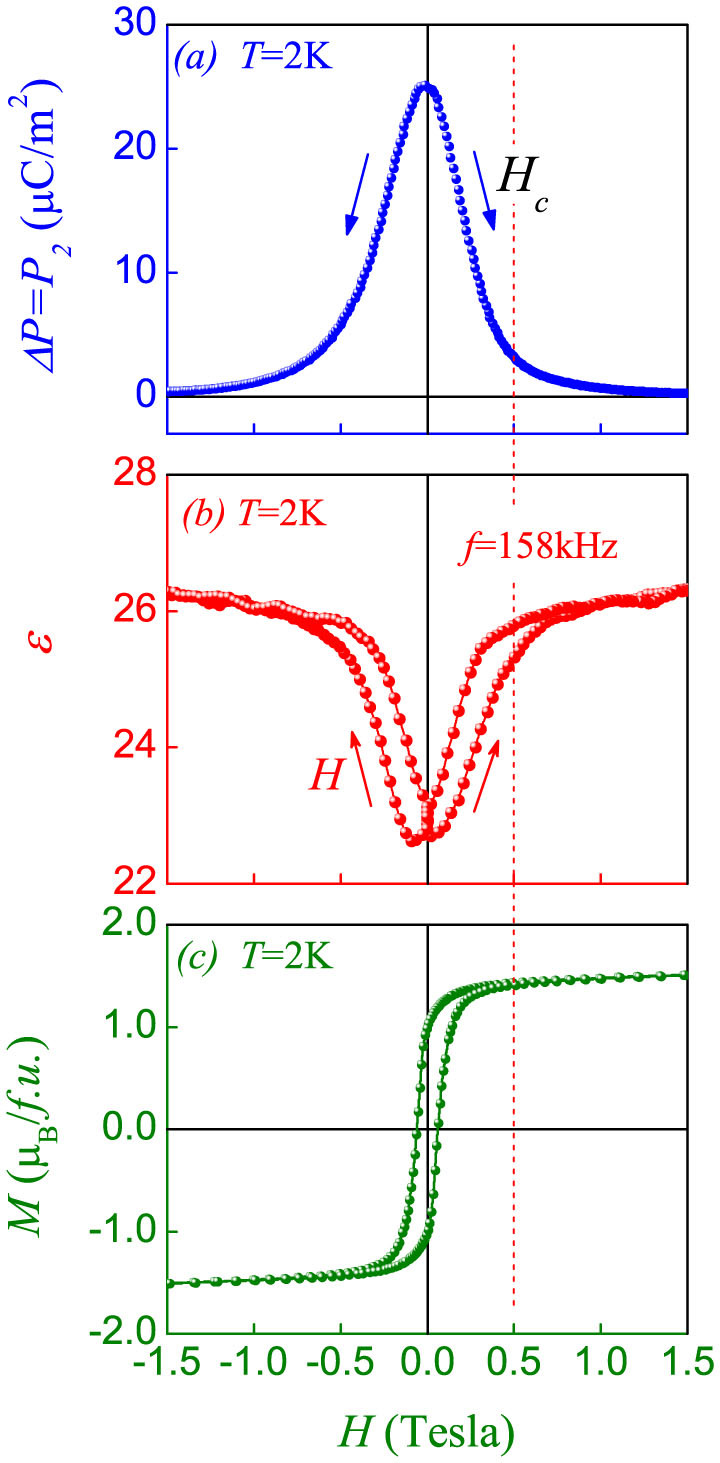
Measured (a) *ΔP* (*P_2_*)-*H* data (b) *ε*-*H* data, and (c) *M-H* hysteresis loop at *T* = 2 K.

Finally, we present the data on the coexistence of ferroelectricity and ferromagnetism. The polarization *P* (Δ*P*), dielectric constant *ε*, and magnetization *M* as a function of cycling *H* at *T* = 2 K are plotted in Fig. 5(a)~(c). The polarization is presented by its variation against *H* = 1.5T from *H* = 0, and this variation equals approximately to *P_2_*. Magnetic field can not only modulate substantially the polarization but also drive well defined ferromagnetic hysteresis. At *T* = 2 K, the saturated *M* reaches as big as ~1.5 *μ_B_*/*f*.*u*. at a low field of ~0.5 T and the coercive field is only 0.06 T. Although the magnetic state is ferrimagnetic, the magnetization is sufficiently big for a number of magnetic applications.

Although both the *P* and *M* exhibit the one-to-one correspondence in response to *H*, an effective inter-coupling between them remains an issue. In FeCr_2_S_4_, the Fe^2+^ spin sublattice and Cr^3+^ spin sublattice are roughly anti-parallel, where each sublattice has relatively weak non-collinear spin component besides the dominant collinear spin component. The ferrimagnetic *M-H* hysteresis shown in Fig. 5(c) reflects that magnetic field cycling reverses the ferrimagnetic lattice from one direction to the opposite one. Meanwhile, the applied magnetic field suppresses the noncollinear spin component and thus suppresses the as-generated polarization component (*P_2_*).

It should be noted that the above two processes may be coupled to some extent but not be necessarily tightly correlated with each other. When *H* decreases from a sufficiently high value, no identifiable current release is observable. This irreversible *P*-*H* sequence is most likely due to that the magnetic field induced collinear spin structure is frozen at *T* = 2 K and the relaxation time for the noncollinear spin structure is very long. This issue still needs to be investigated further. In short, the coupled ferroelectric polarization and magnetization is realized in FeCr_2_S_4_, as further revealed by the discussion on the magnetic origin of electric polarization.

## Discussion

The above experimental results raise three issues needed to address. First, considering the complicated magnetic structure, what physical ingredients are necessary for FeCr_2_S_4_ to be an insulator and even a ferroelectric? Second, two ferroelectric polarization components are observed, and which obviously have different origins. For component *P_2_*, if the noncollinear spin order and spin-orbit coupling are involved, why the ferroelectric transitions occur at the orbit ordering point *T_oo_* rather than the much higher noncollinear spin ordering point *T_nc_* ~ 50 K? Third, for the component *P_1_*, is it the Jahn-Teller distortion responsible for its generation? If so, the structural origin for this component should be discussed.

### Roles of the spin-orbit coupling

**Figure 6:**
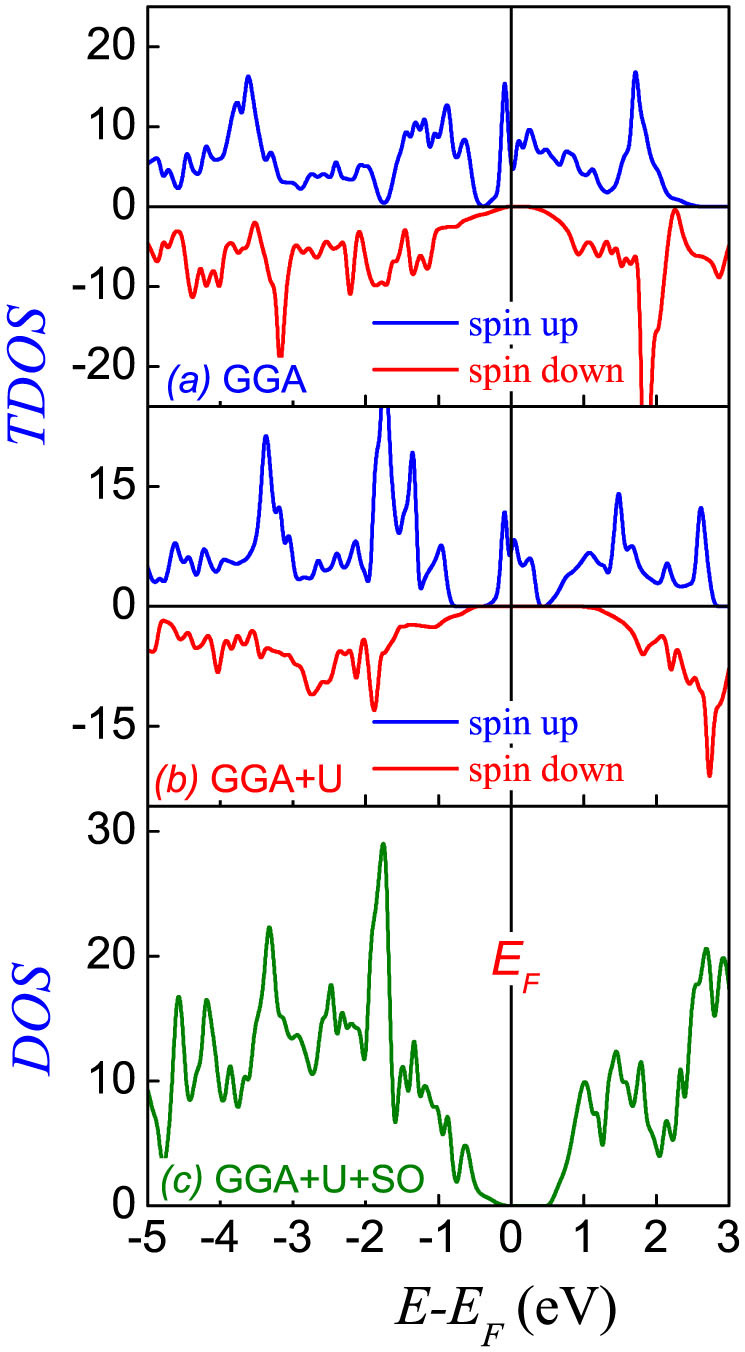
The calculated total density of states obtained for (a) *U_Fe_* = *U_Cr_* = 0 eV (GGA), (b) *U_Fe_* = 5 eV, *U_Cr_* = 2 eV (GGA+*U*), and (c) inclusion spin-orbit coupling (GGA+*U*+SO).

For the first issue, we perform the first-principles calculations on the electronic structure of FeCr_2_S_4_. Although the noncollinear spin order was reported below *T_nc_*, the major spin moments of Fe^2+^ and Cr^3+^ align along the *c*-axis. The calculations in the GGA scheme predict a metallic state, as shown by the total density of states (TDOS) for spin-up and spin down in Fig. 6(a), which is physically unreasonable. The Fermi level always passes through the spin up channel, suggesting the half metal ferrimagnetic behavior. Subsequently, the on-site Coulomb interactions (*U*) are imposed respectively on the Fe^2+^ site and Cr^3+^ site in the GGA+*U* scheme. The calculated TDOS for *U_Fe_* = 5 eV and *U_Cr_* = 2 eV are shown in Fig. 6(b), consistent with earlier report^22^. The half-metal ferrimagnetic states are predicted no matter how the *U_Fe_* and *U_Cr_* are taken in the reasonable region (0.0 ~ 6.0 eV). Although the GGA scheme may underestimate a bit the band gap, the present results suggest that additional ingredient should be included.

Subsequently, the spin-orbit coupling (SOC) effect is considered. Inclusion of the SOC results in weak spin moments on the *ab*-plane in addition to the dominant antiparallel moments along the *c*-axis. Due to the in-plane moments, no TDOS spectra for the spin-up and spin-down bands can be obtained. However, the in-plane moments are too small (~0.01 *μ_B_*) to be considered practically. In Fig. 6(c) is plotted the DOS spectrum, and one observes clearly a band gap of ~0.6 eV once the SOC is included, indicating the substantial role of the SOC in driving FeCr_2_S_4_ into an insulator with direct band gap. Most likely, this gap can't maintain the high temperature ferroelectricity but is sufficient for multiferroicity at low *T*. Besides, the calculated energy *E* in the GGA+*U*+SOC case is −85.56 eV, lower than that within the GGA+*U* (*E* = −83.74 eV), implying that the insulator state is more stable. It is suggested that the SOC is one of the critical ingredients of physics in determining the electronic structure of FeCr_2_S_4_. What should be mentioned here is that the SOC is also a critical mechanism for ferroelectricity generation in a noncollinear spin lattice via the asymmetric exchange striction, to be addressed below.

### Spiral spin structure

**Figure 7:**
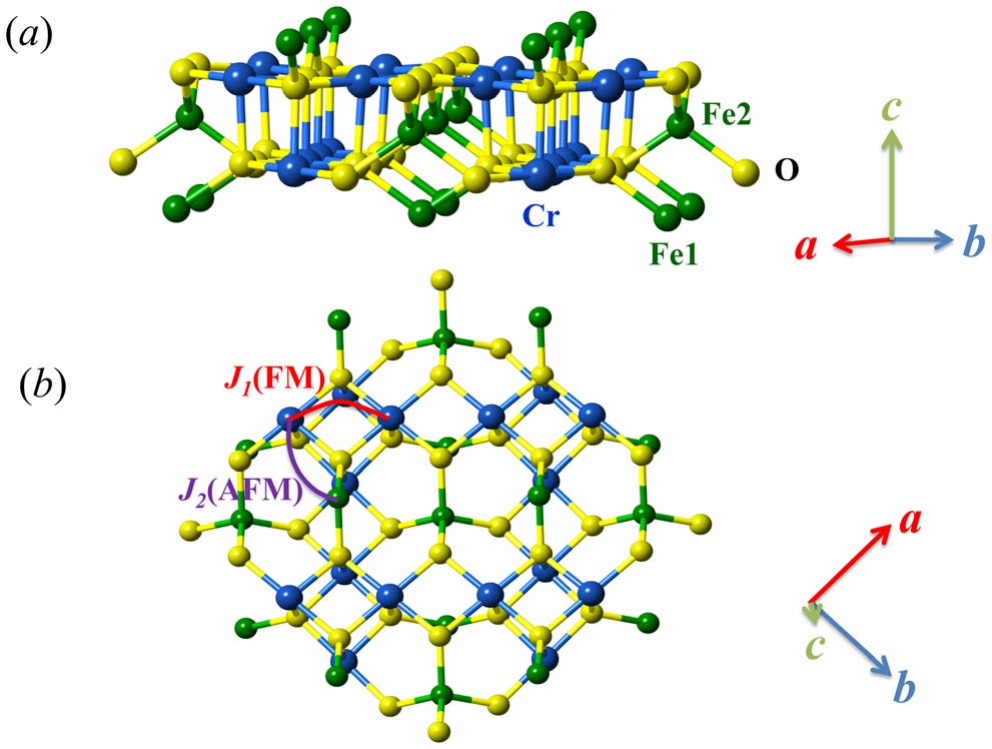
(a) Schematic structure of spinel FeCr_2_S_4_. Each Cr layer is accompanied by up Fe2 layer and down Fe1 layer. (b) Spin-exchange interactions in FeCr_2_S_4_. The nearest neighbor ferromagnetic exchange *J_1_* on the Cr-Cr bond along [110] direction, and the next- nearest-neighbor antiferromagnetic exchange *J_2_* on the Fe-Cr bond are considered.

We deal with the second issue: origin for polarization component *P_2_* which is sensitive to *H*. Since details of the magnetic structure are not available to us, our discussion is qualitative and preliminary. Given the considerable role of the SOC, one may discuss relevant experimental evidence for the noncollinear spin order so that a combination of the SOC effect and noncollinear spin order can be utilized to explain the ferroelectricity generation. Earlier Bessel-type μSR experiments indicated the incommensurate spiral spin ordering established below *T_nc_*. Moreover, the Mossbauer data exclude the magnitude modulation of Fe^2+^ moments, and correlate it with the orientation modulation of these moments^31^. Since the Fe^2+^ and Cr^3+^ ions form staggered layers, as shown in Fig. 7(a), the nearest-neighbor (NN) Cr^3+^-Cr^3+^ distance is 3.534Å along the [110] direction and this interaction *J_1_* is dominated by ferromagnetic (FM) superexchange due to the ~90° Cr^3+^-S^2^-Cr^3+^ bond angle. The next-nearest-neighbor (NNN) superexchange interaction *J_2_* is dominated by antiferromagnetic (AFM) interaction between the Cr^3+^-Fe^2+^ pair, as shown in Fig. 7(b). What should be mentioned is that there are two NNN Fe^2+^ sites at the neighboring layers of each Cr^3+^ layer along the *c*-axis, as indicated by Fe1 and Fe2. This implies that four *J_2_* terms compete with one *J_1_* term, which gives rise to the competing spin frustration. It is known that the magnetic phase transitions in spin frustrated systems are abundant, and noncollinear spiral spin order (SSO) due to the competing interactions are often observed in multiferroics. For the origin of SSO, the *J_1_*-*J_2_*-*J_3_* model along with other models predicts that the magnetic frustration between the NN FM interaction and the NNN AFM interaction is a direct rout to generate the SSO phase^37^,^38^,^39^. Similar to RMnO_3_, FeCr_2_S_4_ has both the NN FM and NNN AFM interactions, and thus may have the SSO phase or spiral spin structure.

Nevertheless, the measured ferroelectric transition occurs far below *T_nc_* ~ 50 K, seemingly inconsistent with the above discussion. The reason may be associated with the particular properties of magnetic ground state in cubic AB_2_X_4_ spinel lattice, as proposed by Lyons, Kaplan, Dwight, and Menyuk (LKDM theory) about 50 years ago^40^. Starting from the classical Heisenberg model and considering only the B-B and A-B NN interactions, one learns that the magnetic ground state is determined by parameter *u* = 4*J_BB_S_B_*/(3*J_AB_S_A_*), where *J_BB_* and *J_AB_* denote the two NN and NNN interactions, *S_B_* and *S_A_* are the moment magnitudes at the two sites. Although the LKDM theory may be over-simplified in this sense, it allows a qualitative prediction of the magnetic ground state. For FeCr_2_S_4_, one has *u* = *J_1_*/*J_2_*. However, it was reported that the short range orbital fluctuations play role in suppressing the magnetic transitions which can't occur unless temperature *T* decreases down to a proper value at which parameter *u*>9/8 for ferromagnetic spiral ordering^23^. Unfortunately, this order results in degenerate spiral spin states. Upon further decreasing of *T*, the cooperative Jahn-Teller distortion becomes no longer negligible and lifts the degeneracy of those spiral spin states so that one of them becomes the ground state. Consequently, one observes the polarization component *P_2_* associated with the SOC driven anionic displacement right below *T_OO_*.

Additional evidence on the above scenario is given by Monte Carlo simulation^41^, in which the role of magnetic anisotropy is addressed, and magnetic anisotropy could stabilize the spiral spin order, leading to a proper *u* value for the spiral spin order. However, the A-A (Fe^2+^-Fe^2+^) exchange interaction enhances the frustration and thus suppresses the spiral spin order. A large magnetic anisotropy was reported in FeCr_2_S_4_^42^. The first-principles calculations also reported that the spin quantization of FeCr_2_S_4_ is along the [001] and [110], while the [110] quantization is favored when the magnetic anisotropy is ~10 meV/Fe and higher^22^. Therefore, all the related evidences indicate a complex magnetic phases under the influence of magnetic anisotropy and exchange coupling.

**Figure 8:**
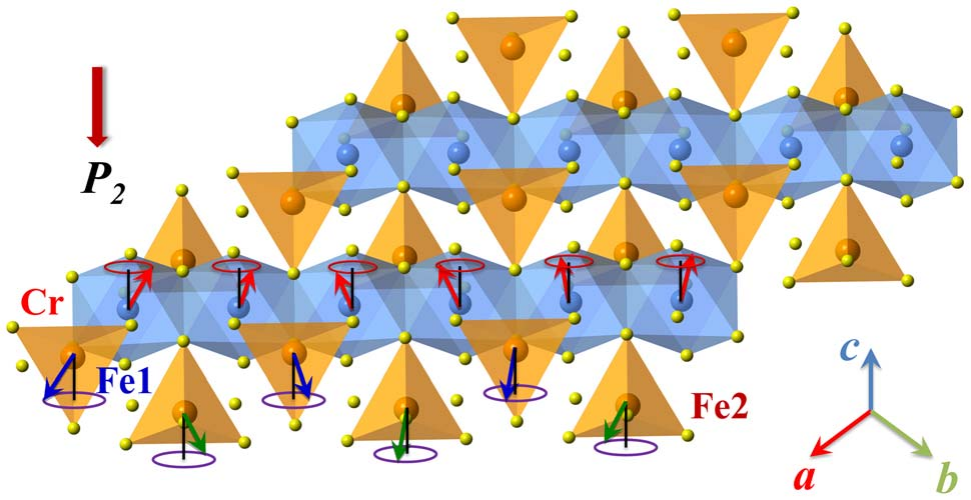
Proposed conical spin order of Cr^3+^ and Fe^2+^ ions in FeCr_2_S_4_. The spin projected on the *ab*-plane generates macroscopic polarization *P_2_*, while the magnetic moment along the *c* direction forms the ferrimagnetic Fe^2+^ and Cr^3+^ sublattices.

Based on the above scenario, a straightforward schematic spin configuration is proposed in Fig. 8. Both the Cr^3+^ and Fe^2+^ spins form the conical spiral spin order below *T_oo_*. The circles and slanted arrows represent the spiral plane of the respective spins. It can be seen that the Cr^3+^ and Fe^2+^ moments align mainly along the *c*-axis with antiparralle arrangement. On the *ab* plane, the Fe1 (Fe2) and Cr spins rotate to form a transverse spin spiral propagating along the (-110) direction. The generated polarization can be explained by the antisymmetric magnetostriction: 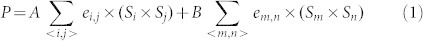
where A and B denote the coupling constants determined by the SOC and Fe^2+^-Cr^3+^ exchange. The unit vector *e_i,j_*and *e_m,n_* connect two Fe spin sites *i*, *j* and two Cr spin sites *m* and *n*. It is thus suggested that the two types of canted spins can generate an electric polarization via the SOC, and the electric polarization (*P_2_*) prefers to align along the [110].

### Jahn-Teller distortion

Finally, we discuss the possible origin for the polarization component *P_1_* appearing at slightly lower *T* than *T_oo_*, which is insensitive to magnetic field *H*. One has reason to argue that it is induced structurally rather than magnetically. In fact, early high-resolution X-ray experiment showed a considerable broadening of the diffraction line that sets in around *T_nc_*, which reaches a peak at *T_oo_*^23^. This evidences a structural anomaly at the orbital-order transition due to a static cooperative Jahn-Teller effect, implying the strong SOC role. In addition, the thermal expansion^23^, ultrasound spectroscopy^43^, and Raman-scattering data^32^ indicate the lattice anomaly near *T_oo_*.

Roughly speaking, the Jahn-Teller distortion on one hand further splits the degeneracy of Fe^2+^ *e_g_* doublet and gives rise to the orbital ordering transition, suggesting the mutual interaction between spin configuration and orbital correlations^23^. On the other hand, based on the first-principles calculation^22^, the crystal symmetry reduction from the *Fd*3*m* to *F*-43*m* is related to the combined effects of tetragonal distortions due to the Jahn-Teller active Fe^2+^ ions and trigonal distortions due to a minor displacement of Cr^3+^ ions along the [111] direction^22^. Under a small Cr^3+^ displacement, the S^2−^ movement tends to create two inequivalent FeS_4_ tetrahedra with two different volumes, which dominates the experimental observed lattice distortion. Besides, it is also reported that the S^2−^ movement prefers to increase the band gap, while Cr^3+^ ion movement tends to narrow it^22^. The *T*-dependent displacement parameter of S^2−^, Fe^2+^, and Cr^3+^ ions are presented by high-resolution X-ray experimental data, with larger displacement of S^2−^ and the smallest displacement of Cr^3+^, which implies the intense correlation between JT distortion and band gap or even ferroelectricity^23^. Therefore, the as-generated polarization, if any, would be gradually enhanced with decreasing *T*, as shown in Fig. 4(c).

It should be mentioned that the Jahn-Teller distortion induced ferroelectricity was proposed for quite a few displacive-type normal ferroelectrics and relevant theoretical consideration was given by Bersuker^28^,^44^. Although details of the mode-softening with this polarization component remain unclear, our discussion deserves for future investigation.

In conclusion, we have demonstrated the coupled ferroelectricity and ferrimagnetism in magnetic spinel FeCr_2_S_4_. The spin-orbit coupling plays an important role in the formation of ferroelectricity. Due to spin-orbit coupling and noncollinear spiral spin order, FeCr_2_S_4_ evolves into ferroelectric phase at the Fe^2+^ orbital ordering termperature *T_oo_* = 8.5 K. Such a ferroelectric phase on one hand originates from the spiral spin structures of Fe^2+^ and Cr^3+^ sublattices, which are established from the spin frustration and magnetic anisotropy. On the other hand, the Jahn-Teller distortion tends to induce ionic displacement, giving rise to the large band gap and polarization along with the orbital ordering of Fe^2+^. Therefore, our data on FeCr_2_S_4_ evidence the coupling between ferroelectricity, orbital ordering, Jahn-Teller distortion, and magnetism. A preliminary report of the multiferroic data on FeCr2S4 was presented at the 2013 APS March meeting (http://meetings.aps.org/link/BAPS.2013.MAR.F21.1). While this article was proof-read, it became aware to us that Bertinshaw, J. et al reported relevant experiments on possible multiferroic ground state of FeCr2S4^24^.

## Methods

Polycrystalline FeCr_2_S_4_ samples were prepared by conventional solid state reaction method from high purity iron (99.9%), chromium (99.9%), and sulfur (99.9%) powder by thoroughly grinding and then sealing in evacuated quartz tube. The samples were slowly heated to 1000°C and iso-thermally annealed for about one week. It is noted that a stoichiometric Fe:Cr:S mixture is practically not sufficient for sintering stoichiometric FeCr_2_S_4_ samples since the as-synthesized powder contains tiny Cr_2_S_3_ impurity phase. In order to exclude this impurity phase, slightly excess Fe and S powder was added. Subsequently, the as-sintered powder, containing FeS impurity rather than Cr_2_S_3_ impurity, was washed to remove the FeS impurity, leaving pure FeCr_2_S_4_ powder. Finally, the powder was re-ground, pressed into pellets, and sintered in evacuated quartz tube at 1000°C for about 3 days to obtain the high dense ceramic disks. The phase structure was checked by X-ray diffraction (XRD) (Bruker Corporation) equipped with Cu *K_α_* radiation. It is noted that we also grew FeCr_2_S_4_ single crystals using the flux solution method. The crystals are ~0.20 mm in size and contain impurity phase which excludes reliable electrical measurements. Therefore, we only present our data on the polycrystalline samples.

For magnetic properties, the *dc* magnetization (*M*) as a function of *T* under the zero field cooled (ZFC) and field cooling (FC) sequences respectively was measured by the superconducting quantum interference device magnetometer (SQUID) (Quantum Design, Inc.), with the cooling and measuring magnetic field of 50 Oe. The isothermal magnetic hysteresis (*M*. vs. magnetic field *H*) was also measured. The specific heat *C_p_* was measured using the standard thermal unit with the physical properties measurement system (PPMS) (Quantum Design, Inc.).

For electrical measurements, the disk-like samples of 6.0 mm in diameter and 0.12 mm in thickness were deposited with Au electrodes on the top/bottom surfaces. The dielectric constant *ε* as a function of *T* was measured using the HP4294A impedance analyzer attached to the PPMS. The equivalent parallel circuit model can fit the data well, in which the accuracy is less affected by leakage current. Voltage dependence of the permittivity was also performed to rule out a Schottky diode like character. In our experiments, for each measurement we used both Au and silver paste electrodes and didn't observe remarkable difference in terms of the dielectric constant and pyroelectric current. The ferroelectric polarization *P* was measured by probing the constant-field pyroelectric current and isothermal polarized current via the Keithley 6514 electrometer connected with the PPMS too. For the pyroelectric current measurement, the sample was electrically poled under an electric field *E_p_* = ±3.8 kV/cm and cooled down from 100 K to 2 K. Then the poling field was removed and the sample was electrically short-circuited for 30 min to several hours in order to exclude possible contributions other than the polarized charges associated with *P*. Usually, this discharging process continues until the background is less than 0.2 pA. The pyroelectric current *I_pyro_* during the subsequent warming process was collected at different ramping rates of 2–6 K/min, respectively.

The ferroelectric hysteresis loop was measured using Positive-Up-Negative-Down (PUND) method. In order to improve the precision of the data read-out, Keithley 6517B electrometer was chosen to output a point by point triangular voltage, e.g., each triangular wave width is ~ 8.5 s. In our practical measurement, a voltage is first applied on for a specific time, then the current is read out. Such delay time is fixed and reliable. The first positive and negative triangular voltage are used to fully align the ferroelectric domains. Then, during the next two positive (negative) ones, two effective polarization curves are recorded. A subtraction of the two sets of current data gives the pure polarization.

For the pyroelectric current (*I_pyro_*-*T*) measurements under a constant *H*, the assigned field *H* was applied to the sample at *T* = 2 K before the electric short-circuiting process until the end of the measurements. However, for the isothermal polarized current (*I_pole_*) probing against *H* (*I_pole_*-*H*), the sample was first electrically poled and cooled down to an assigned *T* in the FE phase with *H* = 0, and then the electric field was removed, followed by sufficiently long time electric short-circuiting. The released *I_pole_* upon increasing *H* from *H* = 0 to *H* = ±4.0 T at a rate of 100 Oe/s was collected.

It brings our attention that the electronic structure of FeCr_2_S_4_ is sensitive to the SOC which is also a critical physical ingredient for generating the ferroelectricity. Our pre-calculations indicated that FeCr_2_S_4_ is always a half-metal if the SOC is not included. This stimulates us to focus on the electronic and magnetic structures of FeCr_2_S_4_ by means of the first-principles calculations in complimentary to experimental investigations. However, we have not intended to calculate the ferroelectricity since no specific concrete data on the magnetism (spin configuration) are available.

The first-principles calculations are performed using the Vienna *ab initio* simulation package (*vasp*) code^45^ and the Perdew-Becke-Erzenhof (PBE) generalized gradient approximation (GGA) function^46^. The lattice parameters are first fixed to our refined XRD data based on a cubic symmetry, until an optimized value of 9.8893Å is obtained. Here the cubic lattice symmetry rather than the triclinic symmetry is used since the latter has yet not confirmed, and quite a few earlier reports indicated the quasi-cubic symmetry in the extremely low *T* range^47^. The projector-augmented-wave (PAW) pseudopotential^48^ is utilized with a plane-wave energy cutoff of 500 eV, and we use a 9 × 9 × 9 Monkhorst-Pack mesh of *k* points. In order to include the on-site Coulomb interaction, the GGA plus Hubbard *U* (GGA+*U*) methodology^49^ is used, and *U* is varied independently on both the Fe^2+^ and Cr^3+^ sites. Also the SOC effect on the electronic structure is taken into account in details.

In our calculations, the magnetism is included by imposing the most accepted ferrimagnetic alignment of the Fe^2+^ and Cr^3+^ sublattices along the *c*-axis in the optimized lattice structure, and then making the structure fully relaxed once more. The present calculations only give a qualitative scenario on which our discussion is based, partially due to the absence of data on the spin configuration.

## Author Contributions

J.M.L. and L.L. conceived and designed the experiments. L.L., X.M.J., Z.B.Y. carried out the experiments. Z.R.Y. provided the sample. H.X.Z. performed the first-principles calculations. K.F.W., S.D., and J.G.W. discussed the ferroelectric mechanism. J.M.L. and L.L. wrote the paper. All the authors discussed the results and commented on the manuscript.
